# Modelling Heart Rate Kinetics

**DOI:** 10.1371/journal.pone.0118263

**Published:** 2015-04-13

**Authors:** Maria S. Zakynthinaki

**Affiliations:** 1 Department of Electronics, Technological Educational Institute of Crete, and Applied Mathematics and Computers Laboratory, Technical University of Crete, Chania, Greece

## Abstract

The objective of the present study was to formulate a simple and at the same time effective mathematical model of heart rate kinetics in response to movement (exercise). Based on an existing model, a system of two coupled differential equations which give the rate of change of heart rate and the rate of change of exercise intensity is used. The modifications introduced to the existing model are justified and discussed in detail, while models of blood lactate accumulation in respect to time and exercise intensity are also presented. The main modification is that the proposed model has now only one parameter which reflects the overall cardiovascular condition of the individual. The time elapsed after the beginning of the exercise, the intensity of the exercise, as well as blood lactate are also taken into account. Application of the model provides information regarding the individual’s cardiovascular condition and is able to detect possible changes in it, across the data recording periods. To demonstrate examples of successful numerical fit of the model, constant intensity experimental heart rate data sets of two individuals have been selected and numerical optimization was implemented. In addition, numerical simulations provided predictions for various exercise intensities and various cardiovascular condition levels. The proposed model can serve as a powerful tool for a complete means of heart rate analysis, not only in exercise physiology (for efficiently designing training sessions for healthy subjects) but also in the areas of cardiovascular health and rehabilitation (including application in population groups for which direct heart rate recordings at intense exercises are not possible or not allowed, such as elderly or pregnant women).

## Introduction

An understanding of heart rate kinetics combined with the correct and efficient way of data interpretation and analysis is fundamental not only to our knowledge of cardiovascular health and rehabilitation, but also to fitness, weight management, training methodology and also competitive success in sport and exercise. The interest of the scientific community in the area of exercise physiology was originally focused on oxygen uptake kinetics (see for example [1–8]), where the term oxygen uptake (*V̇O*_2_) refers to the product of cardiac output and the volume of oxygen extracted from the blood. As the heart rate is, however, the most commonly used and the easiest to obtain cardiovascular variable, the analysis and modeling of heart time series data recorded during exercise has become an area of major importance.

Let us assume an individual who starts moving from rest, a condition which will be referred to as *movement* or simply *exercise*. The temporal evolution of his/her heart rate (heart rate kinetics) depends on the intensity of the exercise, as well as on a number of other factors, such as temperature, heat, fatigue, age, over-training, nutrition and hydration, altitude, medication, infectious disease or even mental activity [9, 10]. The present work focuses on the effects related to exercise intensity and blood lactate, assuming that all other factors are kept constant.

The mathematical model presented is given as a set of coupled ordinary differential equations in respect to time. Its objective is to describe, simulate, fit existing data and ultimately predict the heart rate response to movement. The proposed model is based on the following requirements:

- A model’s features should reflect the body’s physiological features.
- A model should include as few parameters as possible; the present work assumes only one model parameter, reflecting the subject’s overall cardiovascular condition (which improves with appropriate training).
- The functions forming the model should be smooth functions of time.

In the sections that follow, after a brief introduction to the basic physiological features related to the model as well as the existing models in the area of cardiovascular dynamics, details of the proposed model are provided. The study includes new mathematical models of blood lactate kinetics developed in order to simulate the lactate levels in the blood. Applications of the proposed model to different examples of sets of heart rate data are also presented, together with examples of simulated heart rate kinetics.

### Heart rate kinetics

The maximum heart rate *HR*_*max*_ is the highest value that can be achieved in an all-out effort to the point of exhaustion. It is a highly reliable value that remains constant for a particular subject (see [11] and the references therein) and changes only slightly with age (a slight but steady decrease of about one *beat*/*min* per year, beginning at 10 to 15 years of age, has been observed). Thinking of the heart as a simple pumping machine, it should be expected to have a maximum pumping ability, which will depend on its size, its shape and all its intrinsic mechanical characteristics which make it distinct for each individual. The reduction observed in the values of *HR*_*max*_ seems, along these lines, logical, as all mechanical components of this machine become less efficient as they age. If, for any reason, the heart is called to provide a pumping rate more than the particular maximum value it can afford (this refers to the condition where the heart rate demand is higher than *HR*_*max*_) then it performs at its maximum until it collapses. Thankfully, apart from a heart, humans also posses a nervous system which, at the very dangerous condition of being at, or near, the condition of *HR*_*max*_, interferes by sending all the necessary warnings. This way the feeling of exhaustion saves the heart by pushing the individual to reduce the effort, or simply to stop moving.

Thinking once more of the heart as a blood pumping machine, then the minimum pumping rate will of course be the obvious 0 *beats/min*, a condition which refers to the heart being turned off in the absence of life. As long as there is life and the heart machine is turned on, basic bodily functions require a minimum heart rate in order to be sustained. The resting heart rate *HR*_*min*_ refers to the heart rate at absolute rest, i.e. in the absence of any movement and is measured while the subject is relaxed but awake, in a neutrally temperate environment, and not having recently exerted themselves nor having been subject to stress or even surprise [10, 12, 13]. Professional athletes have been reported to have a resting heart rate as low as 35 *beats/min*, while for a normal person of age 10 years or more there is normally 60 *beats*/*min* < *HR*_*min*_ < 100 *beats*/*min*[9, 14–20]. Men have been shown to have a lower resting heart rate than women with a reported difference of about 5 *beats/min* in athletes.

Generally speaking, a better conditioned heart needs to beat less times per minute in order to maintain the basic bodily functions. The value of *HR*_*min*_ therefore reflects the body’s cardiovascular condition: a decrease in the value of *HR*_*min*_ indicates an increase in fitness (see [11] and the references therein).

Any kind of movement of intensity *v* (“velocity”) imposes a circulatory demand on the body which the heart is called to meet. This demand is in general an intensity and time-dependent function which we will denote as *D*. There is always *D* ≥ *HR*_*min*_. For severe or very high intensity exercise there is *D* ≥ *HR*_*max*_, a condition which in reality means that the heart rate (*HR*) rises abruptly until it reaches *HR*_*max*_, assuming that the exercise can be continued for a sufficiently long time period [6], unless fatigue sets in before *HR*_*max*_ can be achieved. For low intensity exercises there is *D* < *HR*_*max*_. It should be noted here that both *HR* and *D* take discrete values, as they represent the heart rate of an individual, expressed in *beats/min*.

It has been observed (see [21] and the references therein) that for constant and sub-maximal exercise intensities the body’s circulatory demand remains, to a good approximation, constant and equal to the constant value that the heart rate reaches after some time of exercise. This value of the demand can be obtained from the heart rate time series and is a function of exercise intensity only. This special case of constant intensity exercise is very important as it forms the basis for several tests that have been developed to estimate physical fitness [14, 18, 20] and its study is currently the main area of research in exercise physiology.

**Fig 1:**
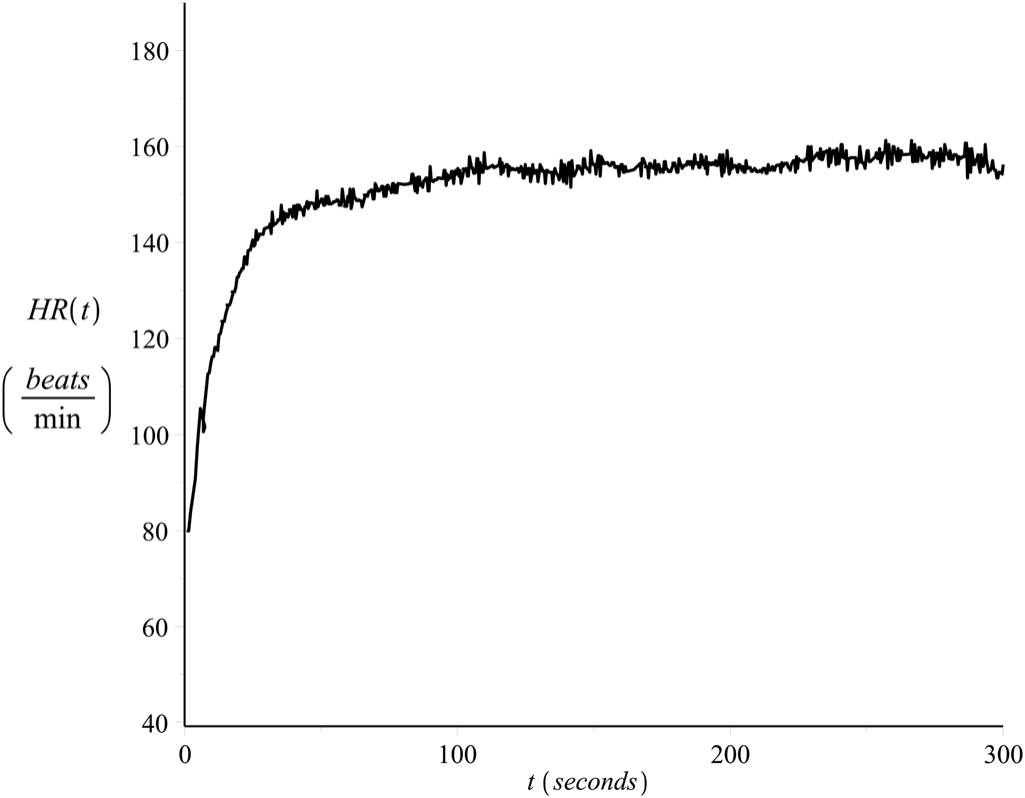
Example of on-transient beat-to-beat heart rate time series. Constant intensity exercise, *v* = 14.4 *Km*/*hr*, please refer to Exercise C in S1 Data Set.

**Fig 2:**
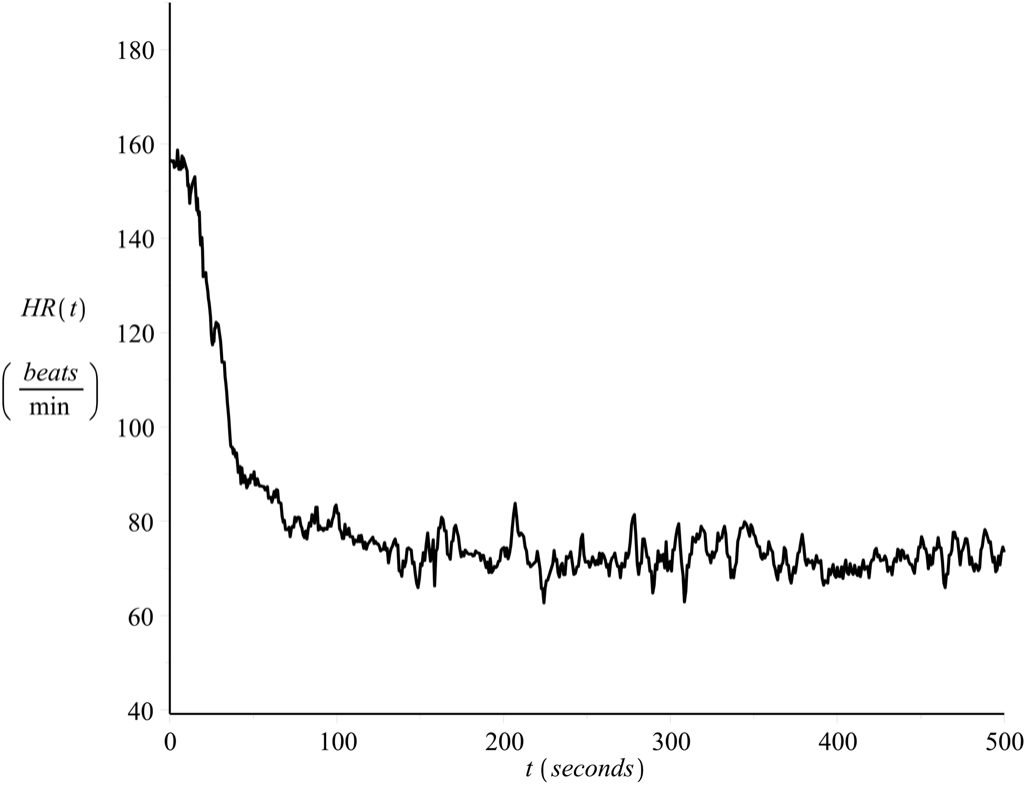
Example of off-transient beat-to-beat heart rate time series. Recovery after the exercise of Fig. 1, please refer to Exercise D in S1 Data Set.

Fig. 1 shows the heart rate values of a 33 year old male runner, recorded on a beat-to-beat basis (see also [22, 23]) during exercise (*on-transient*), please refer to Exercise C in S1 Data Set. The subject’s maximum heart rate value was 185 *beats/min* and the resting heart rate value 40 *beats/min*. The exercise intensity was strictly controlled in order to be kept constant and equal to *v* = 14.4 *Km*/*hr*. Fig. 2 presents the heart rate time series during recovery (*off-transient*) after the exercise of Fig. 1, please refer to Exercise D in S1 Data Set.

It should be noted here that raw heart rate data, recorded on a beat-to-beat basis are necessary for the development of a model: a model which is fit to averaged data is not necessarily a good model of the raw un-averaged data. It is widely accepted that beat-to-beat recordings exhibit spontaneous fluctuations which may have biological significance, as non-linear systems such as the one that governs the circulatory function can produce signals which look like random noise but are in fact not stochastic. Therefore part of what is attributed to noise can contain inherent features and vital information (see [31] and the references therein).

### The effects of blood lactate accumulation on heart rate kinetics

The blood lactate is a chemical compound that plays an important role in various biochemical processes. The concentration of lactate in arterial blood when the body is at rest is around *L*_*basal*_ = 1 *mM*. During exercise this value begins to increase, as this ensures energy production so that the exercise can continue [15, 16, 24]. The elevated blood lactate values are responsible for the ‘burning’ feeling in the muscles during exercise. Such values can approximately reach a concentration of *L*_*max*_ = 12 *mM*, a number which varies in the literature as it depends on the type of exercise, see for example [24] or [25].

Lactate testing is very important in exercise physiology because it reveals the range of intensities at which aerobic base training is carried out, allowing a numerical and objective analysis of work output. For this reason an significant lactate value, the so-called lactate threshold, has been defined [26] as the value of exercise intensity at which blood lactate begins to accumulate. This value depends on the physical condition of an individual and corresponds to a blood lactate value of around 4 *mM* [9, 15, 16, 24, 27, 28].

**Fig 3:**
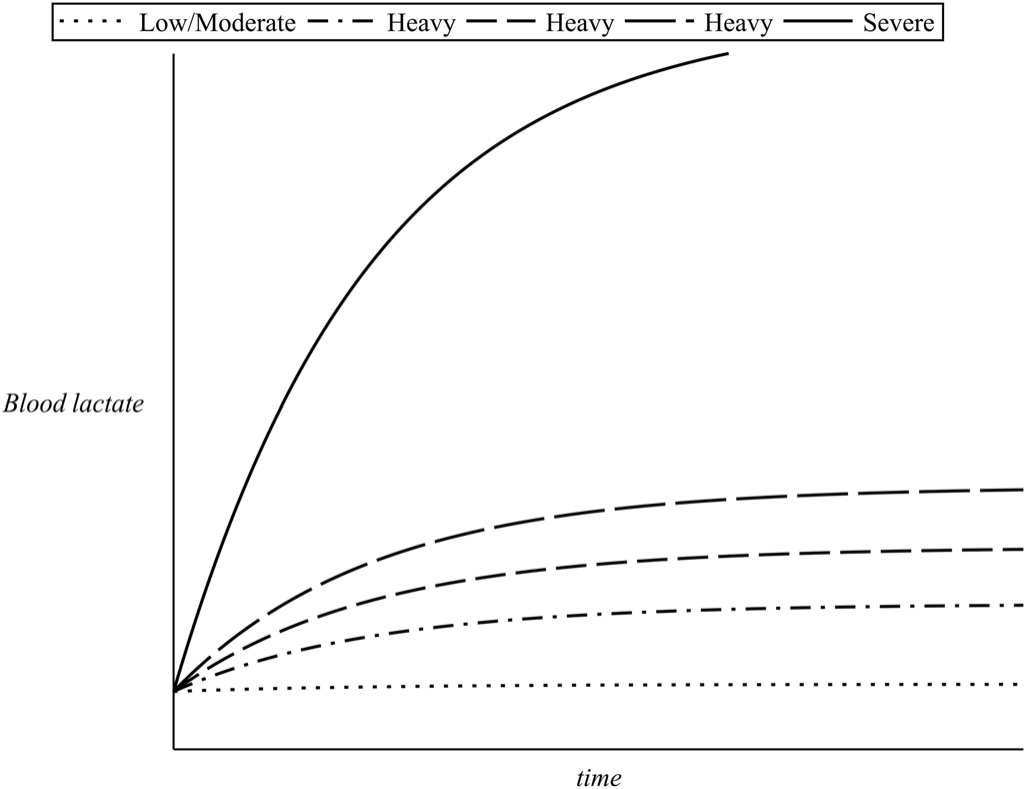
Blood lactate kinetics for different exercise intensities.

During exercise (on-transient) of low or moderate intensity blood lactate are maintained close to their resting levels and the heart rate quickly reaches a steady state which correspond to the demand of the particular exercise intensity (as discussed in the previous section). At heavy exercise intensities around the lactate threshold, the values of blood lactate rise above the resting value and only level off after approximately 10–20 minutes. For such exercise intensities the achievement of a steady state in the heart rate values is delayed: a slow increase in the heart rate values is observed, a phenomenon known as the *slow component* of cardiovascular kinetics, see [6] and the references therein. If the intensity of the exercise is severe, then a steep increase in the blood lactate is observed after the onset of exercise. This increase continues until the individual becomes exhausted and therefore slows down or simply stops. The higher the exercise intensity, the steeper the increase in the blood lactate values. Fig. 3 illustrates the lactate kinetics in respect to time elapsed after the onset of the exercise for a range of exercise intensities. As for such exercise intensities the blood lactate never reaches a steady state, the heart rate also increases steeply and continues to increase beyond the typical time for reaching the steady-state value (until fatigue sets in and the individual lowers the exercise intensity or stops) [8, 28].

**Fig 4:**
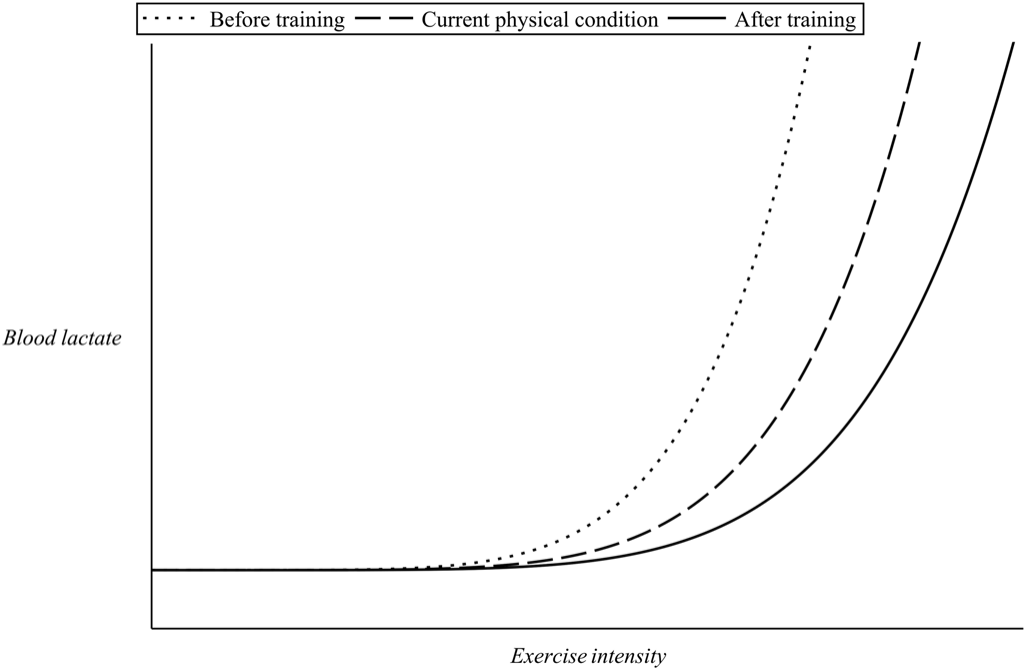
Effect of training on blood lactate.

The observed slowing down in cardiovascular kinetics is a very interesting and important feature in the area of exercise physiology as it has very important applications in the design of training sessions [9, 15, 27–29]. Because of the existence of the slow component, there is a wide range of exercise intensities (which depend on the physical condition of the individual) for which a steady state in the heart rate values cannot be attained. As the slow component is linked to the process of fatigue, the higher the intensity that can be sustained in the absence of slow component, the better the prospects for endurance. Endurance training will have the effect of elevating the value of lactate threshold of an individual and thus eliminate the slow component for some exercise intensities. This way exercise intensities which were initially severe for the particular person might become heavy or even moderate following training [24, 30]. Fig. 4 illustrates this point. Curves similar to the ones shown in Fig. 4 can be obtained via incremental workload test revealing the unique metabolic response to training of each individual subject at a particular level of cardiovascular condition.

**Fig 5:**
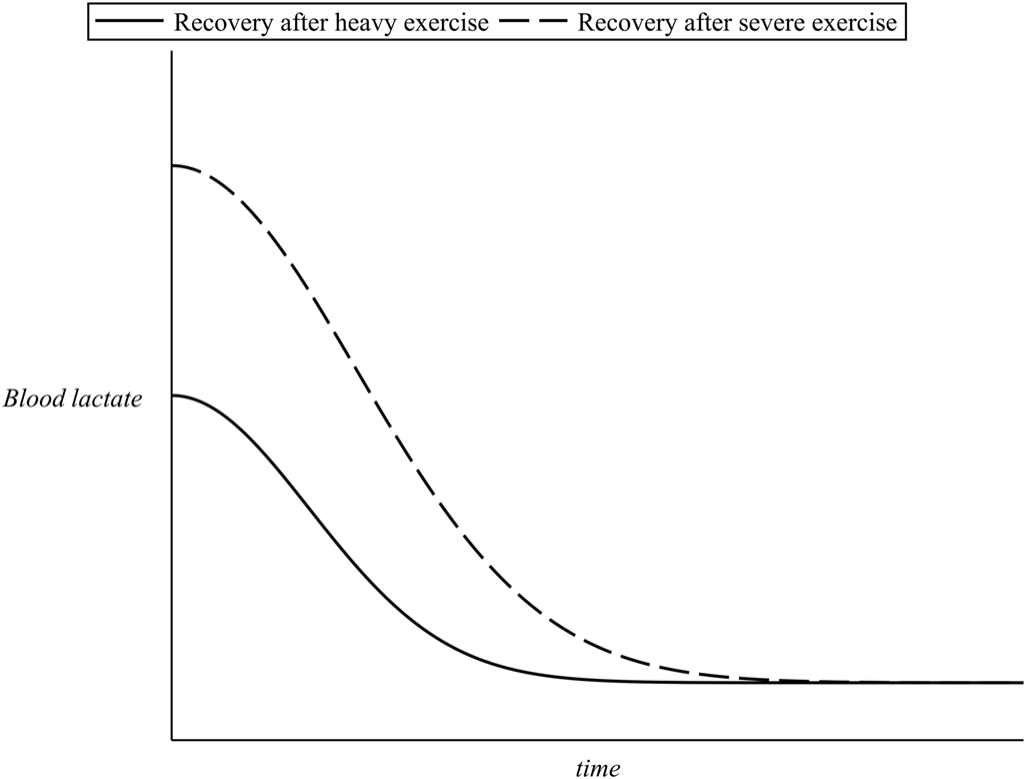
Example of blood lactate kinetics during recovery.

During recovery (off-transient), the initial elevated lactate values produced during the preceding exercise finally return to their resting levels. A certain amount of time is, however, required, in order for the body to clear out any lactate in the blood [9, 16]. The higher the blood lactate values at the termination of exercise, the more the time needed for blood lactate to return to its basal levels (with a time scale of approximately 60 minutes [14, 18, 20, 25]. Fig. 5 presents an example of blood lactate recovery after the end of exercise.

### The 3-phase model

It was originally observed [4] that the kinetics of the cardiovascular variables in response to constant intensity exercise follow an approximately exponential function of time. Later three time-delayed phases were proposed to model the oxygen uptake and heart rate kinetics in response to constant intensity exercise [2, 3, 5, 7, 8, 21].

The 3-phase model has been emerged as the best fit to the data, from a statistical point of view. There is, however, much debate as to its validity: it is very likely that the observed three phases are a figment of the incorrect and overly simple treatment of the data (for a more detailed discussion please refer to [6, 31]). Furthermore, the parameter values that the model provides can only reflect the body’s physiological response to the particular exercise intensity. As far as other exercise intensities are concerned, no reliable predictions can be made. In addition, there is a strong interdependency in the model’s parameters which dramatically affects their values and consequently the confidence of the fitted model.

### Using a set of coupled ODEs to model heart rate kinetics

The model presented in [6, 21] does not assume the existence of phases or time delays. The underlying heart rate kinetics is described as a dynamical system whereby the rate of change of heart rate is assumed to be a function of the current heart rate, exercise intensity and time, so there is $\dot{HR}\equiv \dot{HR}(HR,v,t)$.

$\dot{HR}$ is given as a product of three terms which respectively describe the heart rate kinetics when the value of *HR* is near rest *HR*_*min*_, near maximum *HR*_*max*_ and near the body’s circulatory demand demand *D*(*v*,*t*). Denoting these terms as *f*_*min*_, *f*_*max*_ and *f*_*D*_, then the model is expressed by the following set of coupled ordinary differential equations:

$$\begin{array}{c} \dot{HR}=Af_{min}\left(HR,B\right)f_{max}\left(HR,C\right)f_{D}\left(HR,v,t,E\right) \end{array}$$ (1)

$$\begin{array}{c} \dot{v}=I\left(t\right) \end{array}$$ (2)

where

- *A*,*B*,*C* and *E* are the positive parameters that control the shape of the model curve and provide information regarding the subject’s cardiovascular condition,
- *f*_*min*_ ≡ [*HR*−*HR*_*min*_]^*B*^,
*f*_*max*_ ≡ [*HR*_*max*_−*HR*]^*C*^,
*f*_*D*_ ≡ [*D*(*v*,*t*)−*HR*]^*E*^,
  smooth functions of time and exercise intensity, and
- the vector *v̇* = *I*(*t*) defines the rate of change of exercise intensity, which can be constant (zero exercise intensity refers to absolute rest) or any linear or non-linear function of time (conditions that could not be modelled by use of the 3-phase model).

In the language of dynamical systems [32, 33], the values *HR*_*min*_ and *HR*_*max*_ are repelling fixed points for *HR*(*v*,*t*), as at any time the heart rate tends to move away from these two extremes. The value *D*(*v*,*t*) is an attracting fixed point of the system. The values of the parameters *A*, *B*, *C*, and *E* are very important as they characterize the subject’s current condition across the continuum of possible exercise intensities. Any changes in the parameters indicate changes in the fitness level of that particular subject [6, 11, 21, 22, 34].

The model of equations (1) and (2) has been successfully fit to experimental heart rate time series data, see for example [6, 11, 21, 22, 34]. However, the following weaknesses have been detected:

- Although the parameters *B* and *C* and *E* are introduced in order to control the dynamics in the neighborhood of *HR*_*min*_, *HR*_*max*_ and *D*(*v*,*t*) respectively, careful observation reveals that the terms including these parameters do not become inactive away from *HR*_*min*_, *HR*_*max*_ or *D*(*v*,*t*). As a result the overall rate of change of *HR*(*v*,*t*) is also affected by *B*, *C* and *E*.
- The dynamics of the cardiovascular response to movement is in fact a function of only one parameter, the overall cardiovascular condition of an individual. The model, however, uses the four parameters *A*, *B*, *C*, and *E* instead of a global one.
- Equation (1) does not take into account that the value of *HR*_*min*_ follows the changes in the overall cardiovascular condition.

## Methods

### An improved dynamical systems model

We propose here a modified and improved version of the model of equations (1) and (2).

Following the discussion of the sections above, a model of heart rate kinetics should include only one parameter that reflects the subject’s overall cardiovascular condition. Let us define this global parameter as *λ*; any changes in the value of *λ* will reflect improvements in the overall cardiovascular condition (via appropriate training) or loss (due to lack of training or injury). There will be 0 < *λ* ≤ 1, such that *λ* ≈ 1 refers to an excellent cardiovascular condition.

The proposed model is also expressed in the form of a system of coupled ordinary differential equations, one regarding the rate of change of heart rate and the other regarding the rate of change of exercise intensity. $\dot{HR}$ is expressed again as a product of three terms, *f*_*min*_, *f*_*max*_ and *f*_*D*_, which describe the heart rate kinetics when *HR* is respectively near its resting value, its maximum value, and near the heart rate demand.

The new model is based on the following features:

1. The function *f*_*max*_ that describes the repeller at *HR*_*max*_ is no more assumed to depend on the overall cardiovascular condition, so it does not depend on *λ*. This reflects the fact that the maximum heart rate of an individual is an intrinsic value that does not change with cardiovascular training.
2. The function *f*_*max*_ is not assumed to depend on exercise intensity or time. It is modelled as a function of *HR* only, so there is *f*_*max*_ ≡ *f*_*max*_(*HR*), see Equation (5).
3. Taking into account that the resting heart rate of an individual follows the changes in their cardiovascular condition (see introduction), so that large values of *λ* should reflect low values of *HR*_*min*_, the resting heart rate is modelled as a function of *λ*, see equations (6) and (7).
4. The function *f*_*min*_ that describes the repeller at *HR*_*min*_ is not assumed to depend on exercise intensity or time. It is modelled as a function of *HR* and *λ*, so there is *f*_*min*_ ≡ *f*_*min*_(*HR*,*λ*), see Equation (8).
5. The effects of blood lactate lactate accumulation are also taken into consideration. Recalling the previous discussion, the lactate levels in the blood depend on *λ*, exercise intensity and time. The sections that follow include a detailed discussion on how blood lactate kinetics is modelled in the present study by means of the function *L*(*λ*,*v*,*t*), both regarding on- and off-transient.
6. The heart rate demand is assumed to be a function of *HR*, blood lactate, exercise intensity and time.
7. The heart rate demand is also assumed to be a function of the starting heart rate value *HR*(0), as “memory” effects of previous blood lactate accumulation are also taken under consideration.
  There is *D* ≡ *D*(*HR*(0),*λ*,*v*,*t*), see Equation (10).
8. The function *f*_*D*_ ≡ *f*_*D*_(*HR*,*HR*(0),*v*,*λ*,*t*) that describes the attractor at *D*(*λ*,*v*,*t*) has an extra scaling term that depends on *λ*, see Equation (11).

Taking into account these modifications, the improved model of heart rate kinetics is expressed by the following set of coupled ODEs:

$$\begin{array}{ccc} \dot{HR}\left(HR,HR\left(0\right),\lambda ,v,t\right) & = & f_{min}f_{max}f_{D} \end{array}$$ (3)

$$\begin{array}{ccc} \dot{v} & = & I(t) \end{array}$$ (4)

and will be discussed in detail in the subsections that follow.

### Heart rate kinetics near *HR*_*max*_

As discussed in the previous sections, the body will always try to stay away from the very uncomfortable and crucial condition near, or at, the maximum heart rate. In terms of dynamical systems [32, 33], the repeller at *HR*_*max*_ should therefore be much stronger than the respective repeller described by Equation (1). Furthermore, the body receives warning signs only when the heart rate approaches its maximum values. The respective term of Equation (1) should be therefore modified so as to limit its repelling range inside the neighborhood of *HR*_*max*_.

Assuming that the repeller at *HR*_*max*_ influences an area of a standard deviation *α*_1_ (where *α*_1_ refers to a number of beats per minute), *f*_*max*_ will be assumed to have the form:

$$\begin{array}{c} f_{max}\left(HR\right)\equiv -\left\{1-e^{-{\left(\frac{HR-HR_{max}}{\alpha_{1}}\right)}^{2}}\right\}\;. \end{array}$$ (5)

It is easy to observe that the ODE $\dot{HR}=f_{max}$ has a fixed point at *HR*_*max*_ which has the desired repelling effect, as $\dot{HR}<0$, ∀ *HR* < *HR*_*max*_. Furthermore *f*_*max*_ represents a repeller which, in the neighborhood of *HR*_*max*_, is far stronger than the one used by Equation (1), but has no control on the dynamics away from *HR*_*max*_.

**Fig 6:**
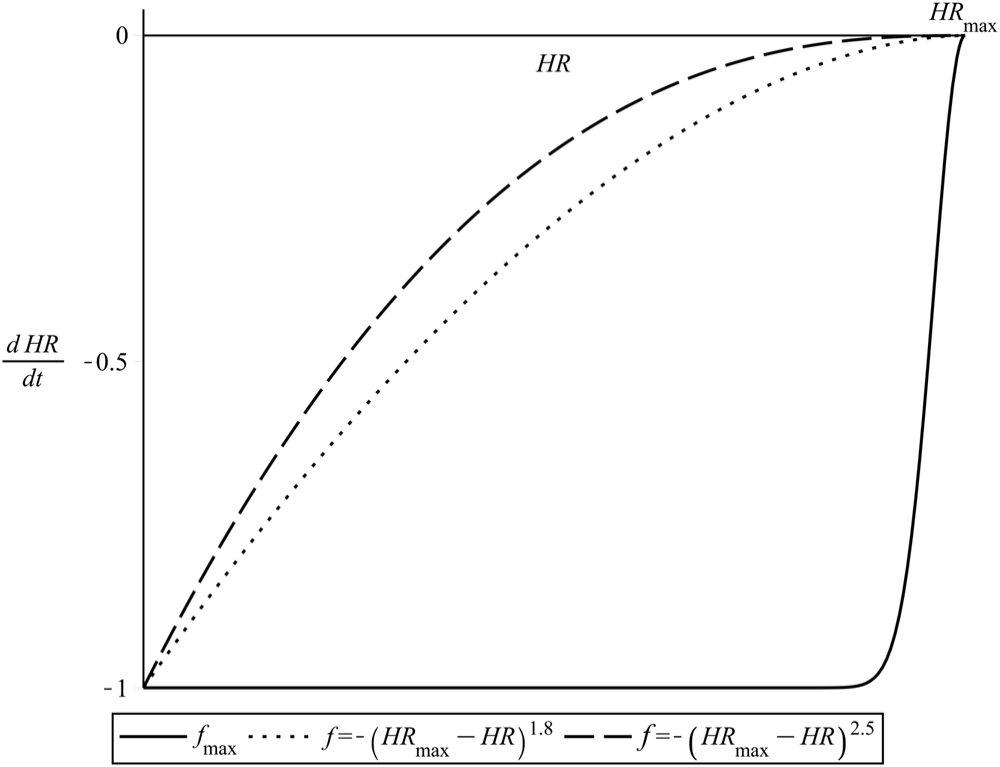
The repelling function *f*_*max*_. Compared to its respective repelling term of normalized Equation (1). *C* = 1.8 and *C* = 2.5.

An appropriate value of *α*_1_ was found, by trial and error, to be *α*_1_ = 10 *beats/min*. Fig. 6 illustrates the repeller given by *f*_*max*_, in comparison also to the respective repelling function of Equation (1) for two different values of the exponent *C*.

### Heart rate kinetics at rest

Assuming that *HR*_*min*_(*λ* = 1) = 35 *beats*/*min* for males and *HR*_*min*_(*λ* = 1) = 40 *beats*/*min* for females (please refer to previous sections), the resting value *HR*_*min*_, for any 0 < *λ* ≤ 1, is modelled as follows:

$$\begin{array}{c} HR_{min}\left(\lambda \right)\equiv \frac{35}{\lambda }\;beats/min\;\;\left(males\right) \end{array}$$ (6)

$$\begin{array}{c} HR_{min}\left(\lambda \right)\equiv \left(\frac{35}{\lambda }+5\right)\;beats/min\;\;\left(females\right). \end{array}$$ (7)

Lowering the heart rate down to values close to *HR*_*min*_ is a real challenge for untrained persons. In the language of dynamical systems, the condition of *HR*_*min*_ is therefore a repeller, which in the present study is modelled as strong as the repeller of *HR*_*max*_. The repelling action of *HR*_*min*_ should also be limited within its neighborhood.

Similar to the case of *HR*_*max*_, *f*_*min*_ will have the form

$$\begin{array}{c} f_{min}\left(HR,\lambda \right)\equiv \left\{1-e^{-{\left(\frac{HR-HR_{min}\left(\lambda \right)}{\alpha_{2}}\right)}^{2}}\right\}\;. \end{array}$$ (8)

where it is assumed that *α*_2_ = *α*_1_ = 10 *beats/min*, as with *f*_*max*_(*HR*).

**Fig 7:**
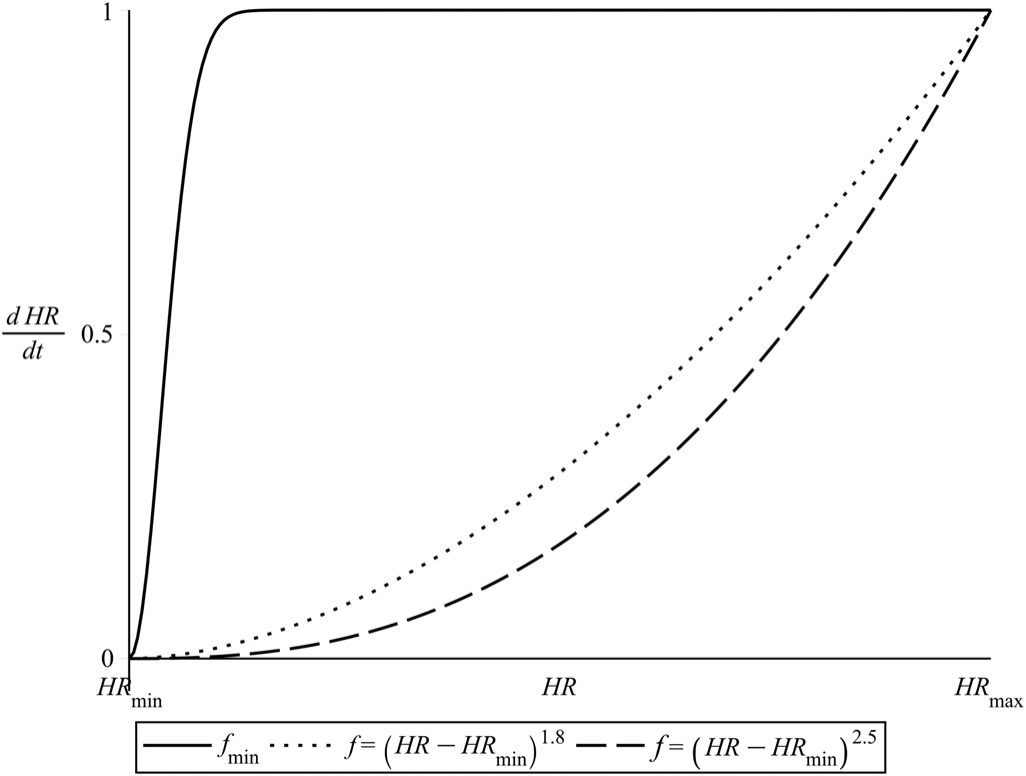
The repelling function *f*_*min*_. Compared to its respective repelling term of normalized Equation (1). *B* = 1.8 and *B* = 2.5.

The point *HR* = *HR*_*min*_ is a repelling fixed point for the ODE $\dot{HR}=f_{min}$, as $\dot{HR}>0$, ∀ *HR* > *HR*_*min*_. Fig. 7 illustrates the above, showing also a comparison of *f*_*min*_ with the repelling function of Equation (1) for different values of the exponent *B*.

As can be seen in Equation (8) the repeller described by the function *f*_*min*_ depends on *λ*, via the dependence of *HR*_*min*_. This way changes in the value of *λ* move the position of the fixed point *HR*_*min*_: if *λ* increases, *HR*_*min*_ moves to the left and vice versa.

### The heart rate demand

As discussed in the previous sections, in the absence of any effects of lactate accumulation (low exercise intensity), the heart rate demand seems to be time-independent. In this case its value, let us call it *D*_*ss*_, is the steady state that the heart rate reaches after a few minutes of exercise. For heavier exercise intensities the fatigue-induced increases in the heart rate values are apparent only after a few minutes after the beginning of the exercise and start to become slowly noticeable (hence the name “slow component”) as the exercise continues.

During passive recovery (complete rest) there is *v* = 0 and the effects of increased body temperature, dehydration, or other unknown factors (which are beyond control, at least for the present study) are apparent and can add up to extra 20 *beats/min*, see also the data shown in Fig. 2).

Defining as *D*_*La*_(*λ*,*v*,*t*) the lactate induced heart rate demand then *D*_*La*_(*λ*,*v*,*t*) will be assumed to be added as an extra component to *D*_*ss*_, simulating slow component effects during on-transient.

Regarding its functional form, *D*_*La*_(*λ*,*v*,*t*) will be assumed to be given both during on-transient and during off-transient by a relation of the form

$$\begin{array}{c} D_{La}\left(v,\lambda ,t\right)\equiv \alpha_{3}L\left(\lambda ,v,t\right)\;, \end{array}$$ (9)

where *L*(*λ*,*v*,*t*) models the lactate values in the blood, for more details see the sections that follow.

The multiplier *α*_3_ > 0 corrects the units and simulates slow kinetics. In the present study the value of *α*_3_ was found by trial and error (during the process of fitting the model to experimental data, see sections that follow) to be equal to *α*_3_ = 4 $\frac{beats/min}{mM}$, both for on- and for off-transient.

### “Memory” effects in heart rate kinetics

Let us assume on-transient (exercise) or off-transient (recovery) starting from an initial heart rate of *HR*(0). As the heart rate changes to reach the demand *D̂*(*λ*,*v*,*t*) ≡ *D*_*ss*_(*λ*,*v*,*t*)+*D*_*La*_(*λ*,*v*,*t*), it is still affected by the attracting heart rate demand of the preceding state. This “memory” effect is far more apparent during off-transient: the attracting effect of the heart rate demand of the preceding exercise does not disappear from one second to the other, instead it slowly and smoothly vanishes to finally allow *D̂*(*λ*,*v*,*t*) to fully drive the kinetics.

The total heart rate demand *D* is assumed to be for a short time “attracted” by the initial heart rate *HR*(0) and to finally reach *D̂*(*λ*,*v*,*t*), following a gaussian-like curve in respect to time. The standard deviation of this gaussian curve is assumed to depend on *λ* and also on how high the initial demand is in comparison to the resting heart rate, i. e. on the difference *HR*(0)−*HR*_*min*_. A relation that adequately describes the above is

$$\begin{array}{c} D\left(HR\left(0\right),\lambda ,v,t\right)=\hat{D}\left(\lambda ,v,t\right)+\left\{HR\left(0\right)-\hat{D}\left(\lambda ,v,t\right)\right\}e^{-\sigma \;t^{2}} \end{array}$$ (10)

where

$$\begin{array}{c} \sigma \equiv \alpha_{4}\lambda {\left(\frac{HR_{max}-HR_{min}}{HR\left(0\right)-HR_{min}}\right)}^{\alpha_{5}}, \end{array}$$

and the multiplier *α*_4_ > 0 was found by trial and error (fit to experimental time series data, see sections that follow) to be equal to *α*_4_ = 0.003 *sec* ^−2^. Similarly the superscript *α*_5_ > 0 was found by trial and error to be equal to *α*_5_ = 4.

### The attracting term *f*_*D*_

Based on the physiological fact that, the better the individual’s physiological condition, the less the time needed to reach the demand, both regarding on-transient and off-transient kinetics [6, 9, 11, 15, 17, 19, 34], the function *f*_*D*_ which will describe the attractor at *D*(*λ*,*v*,*t*) can be defined as follows:

$$\begin{array}{c} f_{D}\left(HR,HR\left(0\right),v,\lambda ,t\right)\equiv -d\left(\lambda \right)\left[HR-D(\lambda ,v,t)\right]\;, \end{array}$$ (11)

where the role of the strictly increasing function 0 < *d*(*λ*) < 1, ∀ 0 < *λ* < 1 is to control the overall dynamics of the kinetics towards the attractor of *D* (please compare also with the parameter *A* that appears in Equation (1)). The functional dependence of *d* on *λ* is assumed here to be linear, thus

$$\begin{array}{c} d(\lambda )\equiv \alpha \lambda \;, \end{array}$$ (12)

where the multiplier *α* > 0 corrects the units and correctly simulates the heart rate kinetics. In the present study (see the numerical sections that follow) the optimal value of *α* was found to be *α* = 0.08 *sec* ^−1^.

The point *HR* = *D*(*λ*,*v*,*t*) modelled as in Equation (11) is an attracting fixed point for the ODE $\dot{HR}=f_{D}$, as $\dot{HR}>0$, ∀ *HR* < *D*(*λ*,*v*,*t*) and $\dot{HR}<0$, ∀ *HR* > *D*(*λ*,*v*,*t*).

### Modeling blood lactate dynamics

We present here the models of blood lactate kinetics used in the present work, both regarding on- and the off-transient.

As was previously defined, *L*(*λ*,*v*,*t*) denotes the function that models blood lactate. Let *L* ^*on*^(*λ*,*v*,*t*) and *L* ^*off*^(*λ*,*t*) refer to on-transient and off-transient respectively (please note that the off-transient blood lactate kinetics is not assumed to depend on exercise intensity *v*, due to the fact that the recovery is assumed to be passive and so *v* = 0).

### Simulating on-transient blood lactate kinetics

As previously discussed, incremental work load tests have revealed that after approximately 30 minutes of steady state exercise the values of lactate in the blood depend on exercise intensity and on *λ* as illustrated in Fig. 4. For heavy exercise intensities the levels of blood lactate follow an approximately exponential rise until a steady state is reached, after approximately 10—20 minutes of exercise, while for severe exercise intensities such a steady state can never be reached as the values of blood lactate continue to rise instead until fatigue sets in (please refer to Fig. 3).

During on-transient, *L* ^*on*^(*λ*,*v*,*t*) is assumed to be a product of two functions, function *L*_*λ*,*v*_(*λ*,*v*) which depends on the level of cardiovascular condition and on exercise intensity, and function $L^{{on}_{t}}(t)$ which is only time dependent:

$$\begin{array}{c} L^{on}\left(\lambda ,v,t\right)\equiv L_{\lambda ,v}\left(\lambda ,v\right)\;L_{t}^{on}\left(t\right)\;. \end{array}$$ (13)

Without loss of generality it can be assumed that an individual of *λ* = 1 can maintain an exercise session of approximately 3–4 minutes at intensities as high as *v*_*max*_ = 20*Km*/*hr* (please note that the exercise intensity can be also given in *Watts* [9, 15, 19, 24, 27]). Taking into account the dependence of blood lactate on exercise intensity *L*_*λ*,*v*_(*λ*,*v*) can be modelled by means of an exponential relation of the form:

$$\begin{array}{c} L_{\lambda ,v}\left(\lambda ,v\right)\equiv L_{basal}+\left(L_{max}-L_{basal}\right)exp\left\{\alpha_{6}*\left[v-v_{max}\left(\lambda \right)\right]\right\}\;, \end{array}$$ (14)

where

- *v*_*max*_(*λ*) refers to the intensity to exhaustion (maximum exercise intensity achievable by an individual of cardiovascular condition *λ*) and
- the parameter *α*_6_ controls the curvature of the blood lactate curve.

**Fig 8:**
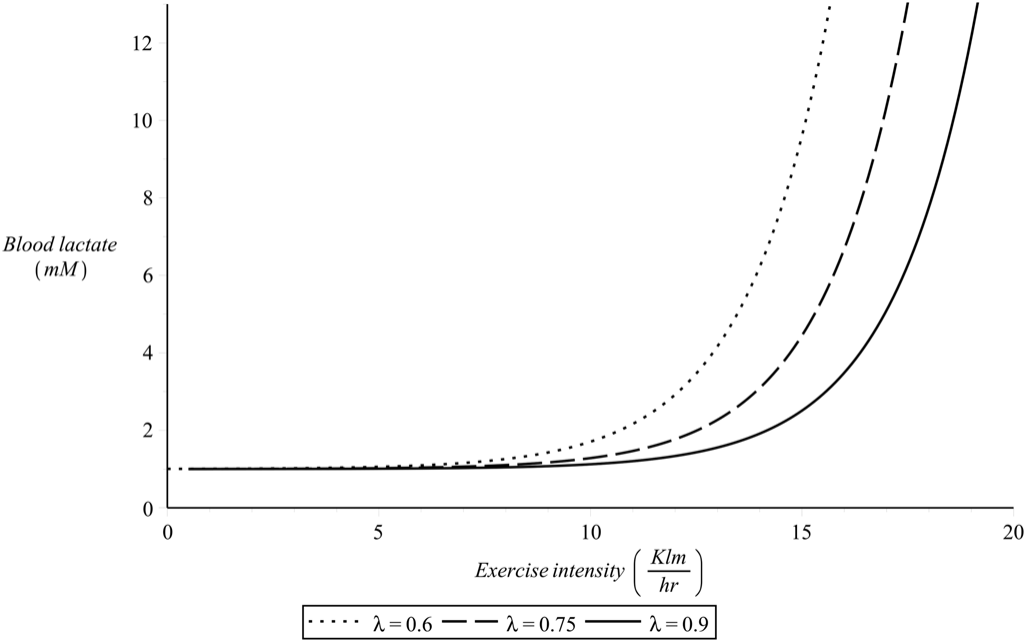
*L*_*λ*,*v*_(*λ*,*v*) as defined in Equation (14) for different values of *λ*. For the numerical simulations there was *α*_6_ = 0.5 *hr*/*Km* and $v_{max}(\lambda )=20\;\sqrt{\lambda }$ (*Km*/*hr*).

The numerical value of *α*_6_ and the functional dependence of *v*_*max*_ on *λ* are to be determined by fit of Equation (14) to experimental data sets of blood lactate in response to exercise intensity. To simulate the dynamics presented in Fig. 4 the value of the parameter *α*_6_ of Equation (14) was chosen here to be *α*_6_ = 0.5 *hr*/*Km* and it is assumed that $v_{max}(\lambda )=20\;\sqrt{\lambda }$ (*Km*/*hr*). Fig. 8 presents examples of this simulation for different values of *λ*.

**Fig 9:**
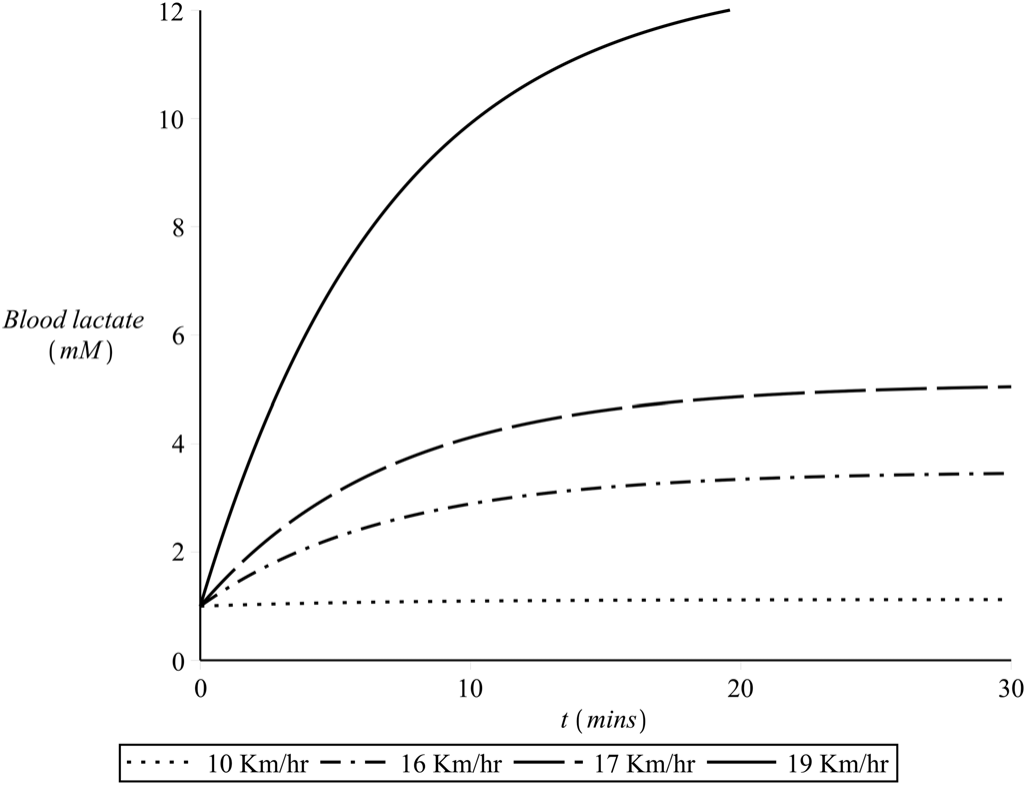
*L* ^*on*^(*λ*,*v*,*t*) as defined by equations (13), (14) and (15) for different values of exercise intensity *v*. For the numerical simulations there was *λ* = 0.9 and *α*_7_ = 420 *sec* ^−1^.

To simulate the time dependence of blood lactate accumulation, the term $L_{t}^{on}(t)$ is assumed to have the exponential form:

$$\begin{array}{c} L_{t}^{on}\left(t\right)\equiv 1-exp\left(-t/\alpha_{7}\right)\;, \end{array}$$ (15)

where the value of the parameter *α*_7_ is again to be determined by fit to time series of blood lactate in response to constant exercise intensity. To simulate the dynamics presented in Fig. 3 the value of the parameter *α*_7_ of Equation (15) was chosen here to be *α*_7_ = 420 *sec* ^−1^. Examples of this simulation for a constant value of *λ* = 0.9 and for different exercise intensities are illustrated in Fig. 9.

### Simulating off-transient blood lactate kinetics

Based on the physiological background previously discussed, the term *L* ^*off*^ should depend on the level of cardiovascular condition *λ*. The time dependency of *L* ^*off*^ is assumed to have the form of a Gaussian bell, with maximum value at *t* = 0 (end of exercise—beginning of recovery) and a standard deviation that depends both on the starting blood lactate value *L*(0) and on the value of *λ*:

$$\begin{array}{c} L^{off}\left(\lambda ,t\right)\equiv L_{basal}+\left[L\left(0\right)-L_{basal}\right]exp\left\{-\frac{\alpha_{8}\lambda }{\left[L\left(0\right)-L_{basal}\right]}t^{2}\right\}\;. \end{array}$$ (16)

**Fig 10:**
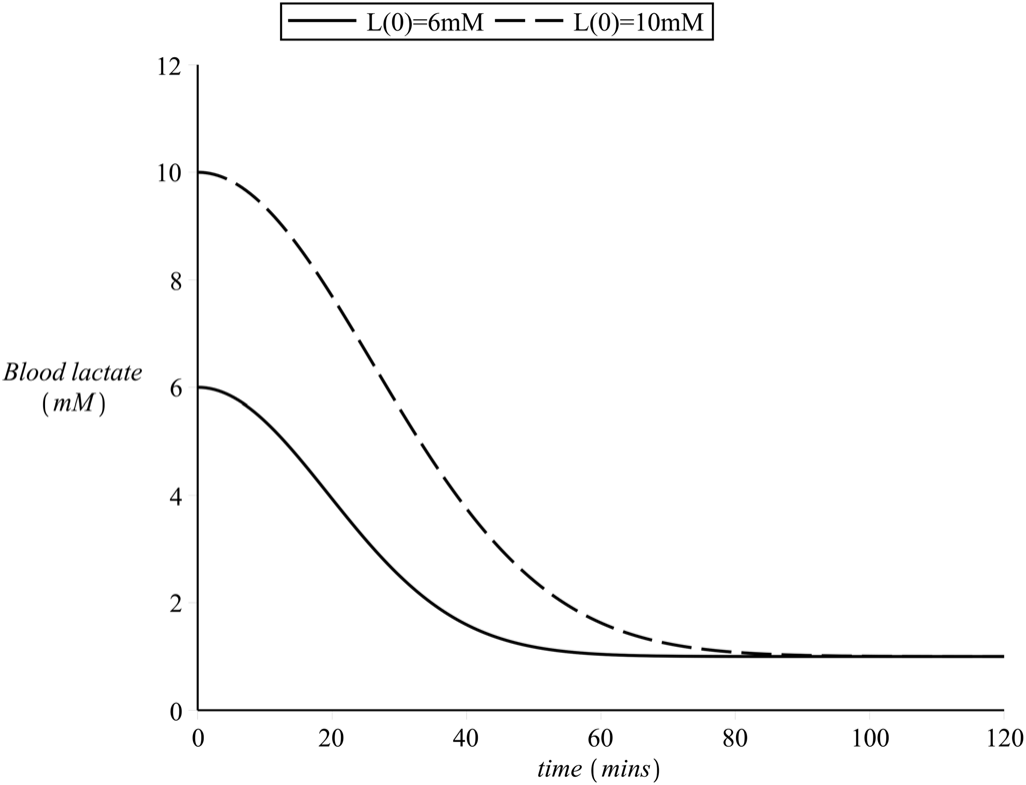
*L* ^*off*^(*λ*,*t*) as defined in Equation (16) for different starting blood lactate values. For the numerical simulations there was *λ* = 0.9.

The value of the parameter *α*_8_ is, as before, to be determined by fit of blood lactate time series during recovery. A value that efficiently simulates the experimental background (Fig. 3) was found to be *α*_8_ = 2.3⋅10^−6^ *mMsec* ^−2^. Fig. 10 presents simulation examples of off-transient blood lactate kinetics.

## Results

### Fit of the model to experimental data

This section presents the numerical application of the proposed model to the experimental heart rate time series data. It should be noted that the model of equations (1) and (2) has been already fit to experimental heart rate data, providing model parameter values for each one of the two case studies [11, 22, 23]. The aim of the present section is to apply the model of Equation (3) to the same data sets in order test the modified model and provide values for *λ*.

The ethics committee of the Department of Physical Activity and Sport (INEF) of the Technical University of Madrid, where the data collection took place (see also [11, 22, 23]) provided an approval for the present study. A written informed consent was signed by the participants before data collection. We confirm that our research meets the highest ethical standards and that it was performed in accordance with the guidelines of the Helsinki Declaration of 1975, as revised in 2000.

As all data sets correspond to constant exercise intensity (see sections that follow) there is *v̇* = 0 and the coupling of the system of equations (3) and (4) disappears. In this special case the heart rate time series data can be modelled by use of Equation (3) only. The numerical solution of Equation (3) was carried out by use of the Runge-Kutta-Fehlberg (RKF45) method [35], with step size set to *h*_*t*_ = 0.01 (the RKF45 is a method of order $𝒪(h_{t}^{4})$ with an error estimator of order $𝒪(h_{t}^{5})$).

As mentioned in the previous sections, the values of *α* in Equation (12), *α*_1_ in Equation (5), *α*_2_ in Equation (8), *α*_3_ in Equation (9), *α*_4_ and *α*_5_ in Equation (10) have been obtained by trial and error during the process of model fitting to experimental time series data, while the values of *α*_6_ in Equation (14), *α*_7_ in Equation (15) and *α*_8_ in Equation (16) have been estimated so as to provide efficient simulations of the existing experimental background. Summarizing, these values are: *α* = 0.08 *sec* ^−1^, *α*_1_ = *α*_2_ = 10 *beats/min*, *α*_3_ = 4 $\frac{beats/min}{mM}$, *α*_4_ = 0.003 *sec* ^−2^, *α*_5_ = 4, *α*_6_ = 0.5 *hr*/*Km*, *α*_7_ = 420 *sec* ^−1^ and *α*_8_ = 2.3⋅10^−6^ *mMsec* ^−2^.

It should be emphasized here that, once calculated, *α* and *α*_*i*_ (*i* = 1,…,8) are fixed numerical values for the model (they are no parameters). In the sections that follow the above shown values of *α* and *α*_*i*_ (*i* = 1,…,8) were fixed for both subjects and all experimental data sets.

The numerical optimization problem consists of finding the optimal value of *λ* that provids optimal fit of the model to the time series data. This value of *λ* is the value that minimizes an appropriately defined cost function which described the problem [11, 22, 23].

Assuming a total number of *N* experimental data points, let us denote as

- ${\left\{HR_{i}\right\}}_{i=0}^{N}$ the experimental heart rate time series and
- ${\left\{HR_{i}^{m}\right\}}_{i=0}^{N}$ the heart rate time series provided by numerical integration of Equation (3).

The cost function *f*(*λ*) can then be defined as the sum of the vertical distances (residuals) between the time series data and the curve provided by the model:

$$\begin{array}{c} f\left(\lambda \right)\equiv -\sum_{i=1}^{N}{\left(HR_{i}-HR_{i}^{m}\right)}^{2}\;. \end{array}$$

The numerical minimization of *f*(*λ*) was carried out the present study by numerical optimization [36, 37] and more specifically by application of the Levenberg—Marquardt algorithm (LMA) [38, 39]. This algorithm, also known as the damped least-squares (DLS) method, interpolates between the Gauss-Newton and the gradient descent methods and is a very popular algorithm used in many numerical applications.

Optimal fit of the model to each time series data provided a small range of optimal values [*λ*−Δ*λ*,*λ*+Δ*λ*] which minimized *f*(*λ*). The final optimal value of *λ* was obtained by the intersection of all optimal *λ* intervals. It should be noted here that the same technique was used in [22] and in [11] to find the optimal parameter values of the model of equations (1) and (2). As the parameters of the model of equations (1) and (2) were, however, three and not just one, these studies considered the intersection of parameter clusters instead.

### Obtaining the value of *λ*: an estimation of the cardiovascular condition of a subject

This section presents the results of the application of the model to sets of heart rate time series of “subject 1”, a 33 year old male runner. The data sets consisted of five on-transient time series corresponding to constant exercise intensity of 13.4 *Km*/*hr*, 14.4 *Km*/*hr*, 15.7 *Km*/*hr* and 17.0 *Km*/*hr*, together with their respective off-transient time series data. Each exercise started from rest and lasted 300 seconds. The subject’s maximum heart rate was 185 *beats/min* and the resting heart rate value 40 *beats/min*.

As the data sets were recorded in one day, allowing appropriate breaks during the recordings, they reflect the cardiovascular condition of the subject for that particular day. For details regarding the data collection protocol please refer to [22] and [23]. Examples of the on-transient and their corresponding off-transient heart rate time series data recorded on that day were shown in Figs 1 and 2.

By numerical fit of the model to the heart rate time series data of subject 1, a small range of optimal values of *λ* was obtained for each data set. The intersection of all optimal *λ* intervals provided the value

$$\begin{array}{c} \lambda_{1}=0.85\pm 0.01 \end{array}$$

as the unique number that reflected the cardiovascular condition of subject 1 during the data recording day.

**Fig 11:**
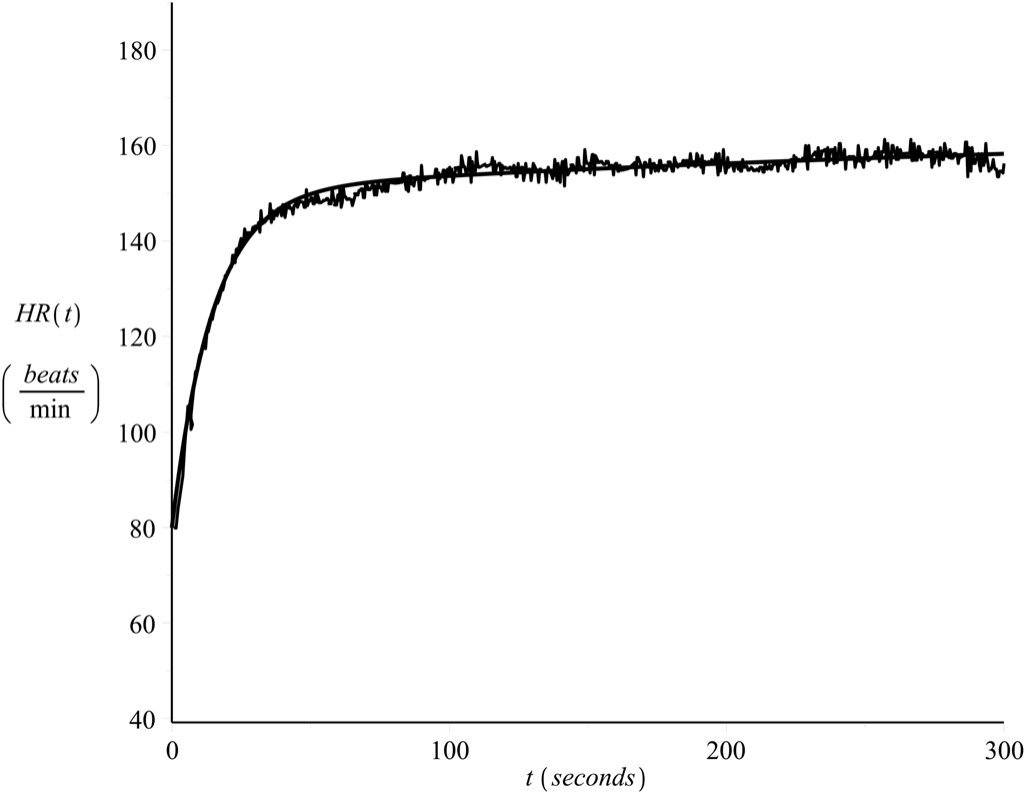
Example of model fit. On-transient heart rate time series, subject 1. *λ* = 0.85.

**Fig 12:**
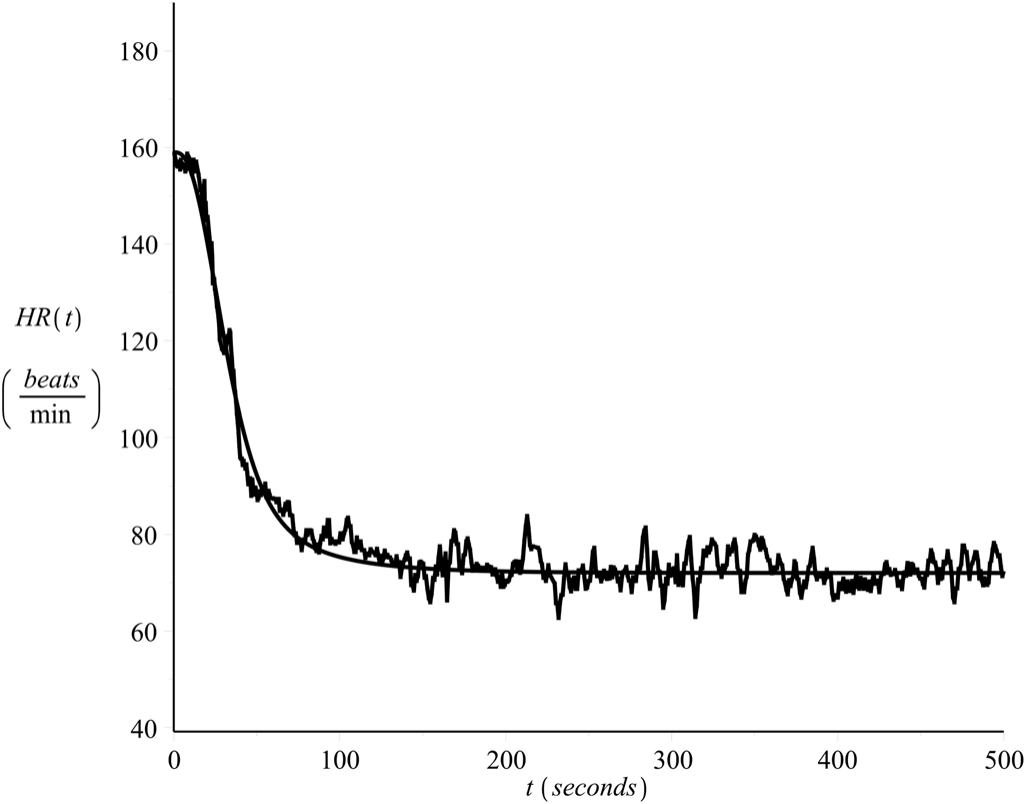
Example of model fit. Off-transient heart rate time series, subject 1. *λ* = 0.85.

Figs 11 and 12 present examples of the fit. The figures show the same data series presented in Figs 1 and 2 together with the curve (solid line) calculated by numerical integration of Equation (1) with *λ* = *λ*_1_ = 0.85. By observation of these figures the success of the model fit is obvious.

### Detecting changes in the cardiovascular condition through changes in *λ*

This section presents the results of the application of the model to sets of time series data of “subject 2”, a 36 year old female before, during and after gestation. The objective was to to detect possible changes in the maternal heart rate kinetics during the different stages of gestation and after labor.

Before gestation the subject’s maximum heart rate was measured to be *HR*_*max*_ = 195 *beats*/*min*. This value has been reported to remain unchanged during gestation [11] and the references therein. The subject did not follow any training during the data recording period; any changes in her cardiovascular condition are therefore due to the body’s adaptation to gestation and are not related to any cardiovascular conditioning from exercise.

The data sets include three on-transient heart rate time series corresponding to low intensity exercise of approximately four minutes of different constant intensities and their respective off-transient time series. For details regarding the data collection protocol please refer to [11]. The subject’s heart rate responses were recorded during a period of approximately two years, and more specifically on the days shown in table 1. Table 1 also shows the subject’s minimum heart rate values at each recording session.

**Table 1:** Subject 2, data details.

**Time**	**Measured *HR*_*min*_**	**Optimal *λ*_2_**	**Estimated *HR*_*min*_**
***(days)***	***(beats/min)***	**(±0.01)**	***(beats/min)***
47 before gest.	61	0.62	61
44, gestation	63	0.60	63
101, gestation	67	0.57	66
163, gestation	73	0.52	72
199, gestation	74	0.51	74
229, gestation	74	0.51	74
262, gestation	74	0.51	74
43 after labour	66	0.58	65
369 after labour	61	0.62	61

Data recording periods, measured resting heart rate, optimal *λ* values and estimated resting heart rate.

Following the same numerical procedure, a value of *λ*_2_ was obtained for each of the data sets of subject 2 (table 1). Based on the calculated values of *λ*_2_, conclusions can be drawn regarding changes in the overall cardiovascular condition of the particular subject during gestation (for a comparison please refer to the study of [11]). As can be observed, one year after labor the subject had returned to her initial cardiovascular condition. Considering the lack of cardiovascular training during the data recording period, this conclusion can provide very important information to the physiologists regarding the beneficial cardiovascular side-effects of pregnancy. Future research can confirm and expand this result.

### Predicting the value of resting heart rate

It is worth observing that, once the value of *λ* is known, the subject’s resting heart rate can be easily estimated by means of equations (6) and (7). For example, the value of *λ*_1_ = 0.85 found for subject 1 provides an estimation of his resting heart rate *HR*_*min*_ = 41 *beats/min* which deviates only 1 *beat*/*min* from the experimentally recorded value.

The estimated resting heart rate values for the different recording sessions of subject “2”, obtained from the optimal values of *λ*_2_ by use of Equation (7) are shown in table 1.

### Using the model to simulate heart rate kinetics

With the values of *α* and *α*_*i*_ (*i* = 1,…,8) kept fixed as before, Equation (3) was numerically solved for a number of hypothetical cases of constant intensity exercise, to provide examples of heart rate kinetics simulations.

**Fig 13:**
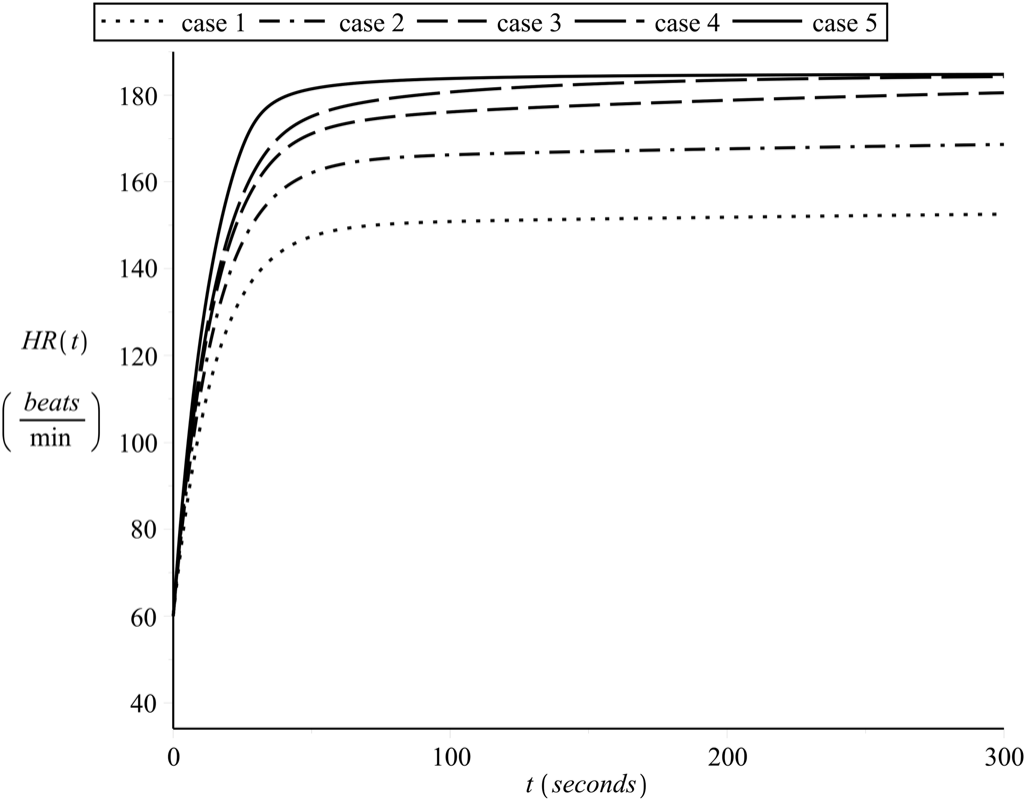
Simulating on-transient heart rate kinetics a. Different constant exercise intensities, starting from the same heart rate. *λ* = 0.85.

Fig. 13 shows examples of simulated on-transient heart rate kinetics for subject 1 (*λ* = 0.85, *HR*_*min*_ = 40 *beats/min* and *HR*_*max*_ = 185 *beats/min*) for different constant exercise intensities, starting from the same heart rate *HR*(0) = 60 *beats/min*. The exercise intensities for the curves shown in Fig. 13 are, case 1: 13.5 *Km*/*hr* (low), case 2: 15.5 *Km*/*hr* (moderate), case 3: 17.0 *Km*/*hr* (heavy), case 4: 18.0 *Km*/*hr* (heavy) and case 5: 19.0 *Km*/*hr* (severe). In Fig. 13 the slow component effect can be clearly observed in the curves which correspond to heavy/severe exercise intensities.

**Fig 14:**
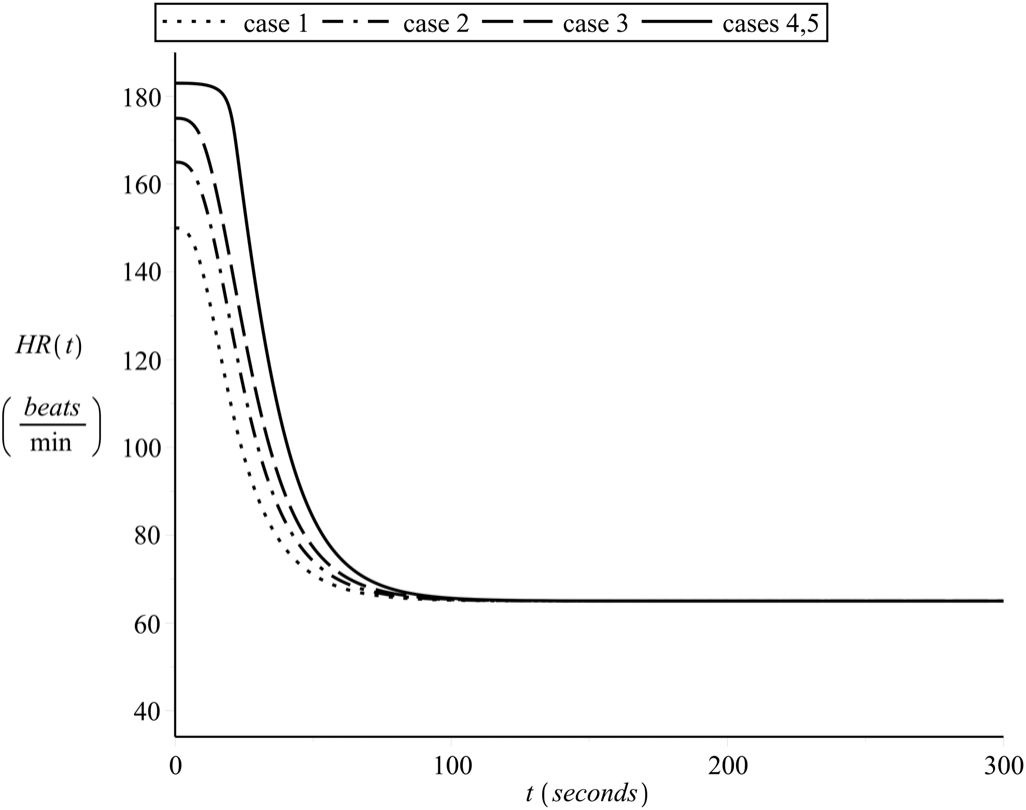
Simulating off-transient heart rate kinetics a. Starting from the end of the on-transient shown in Fig. 13.

The respective simulated off-transient curves are shown in Fig. 14.

### Simulating the effects of changes in *λ*

**Fig 15:**
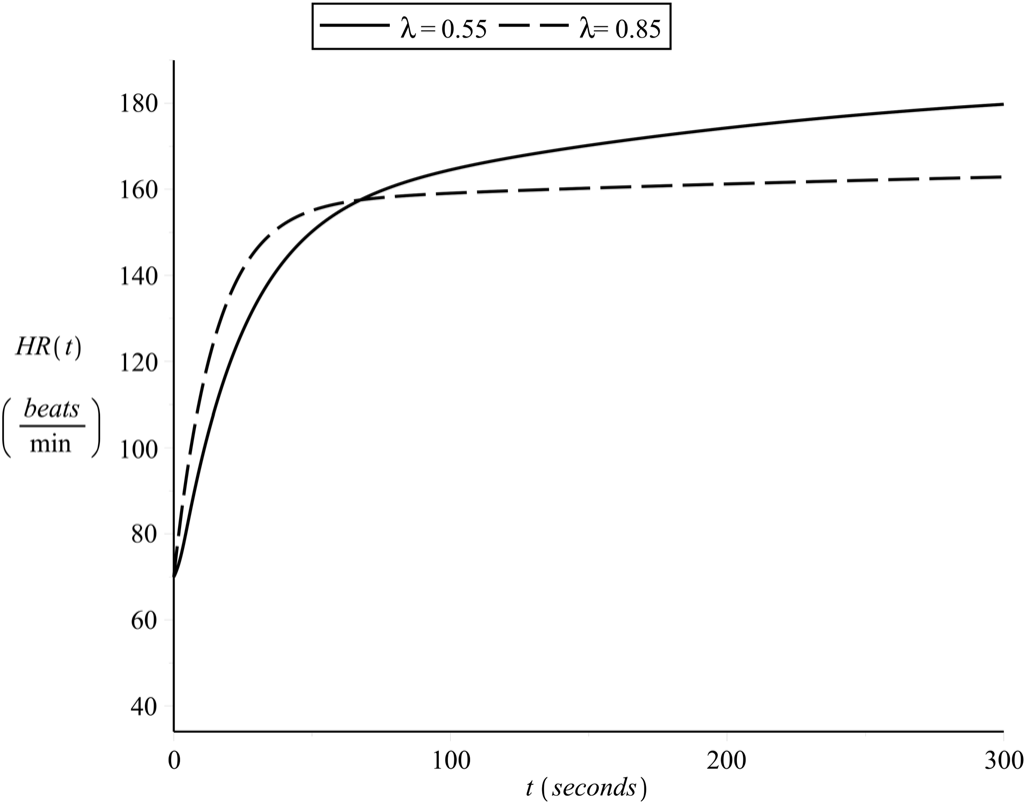
Simulating on-transient heart rate kinetics b. Constant exercise intensity, different values of *λ*.

**Fig 16:**
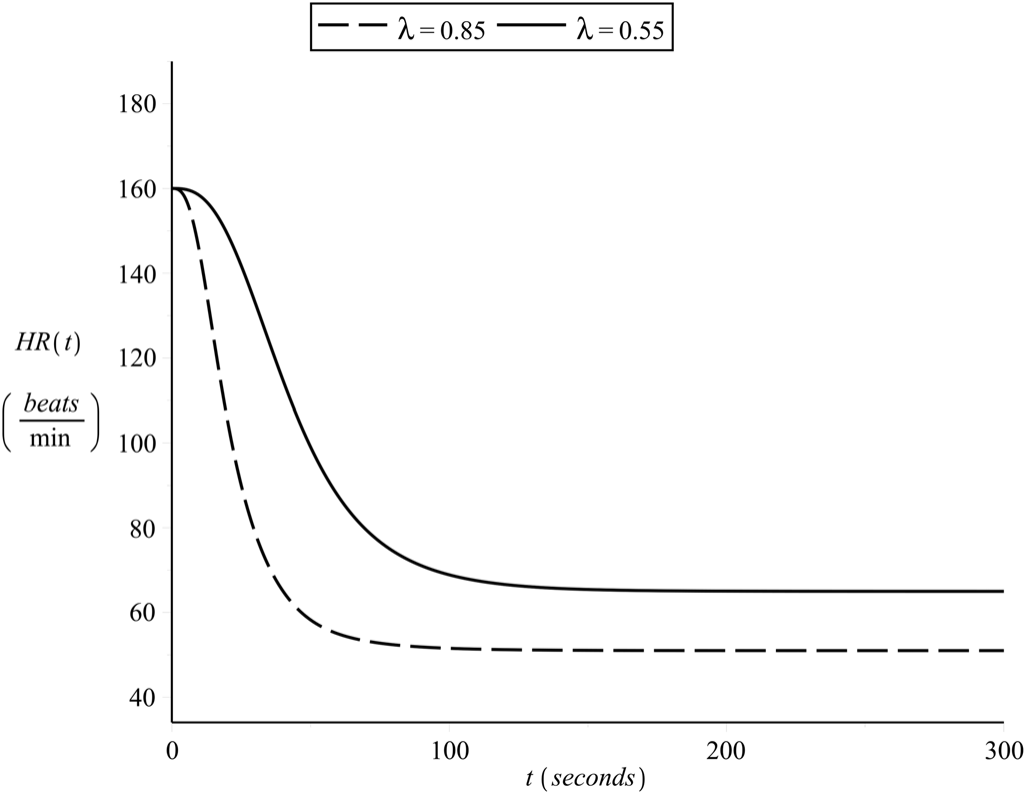
Simulating off-transient heart rate kinetics b. Constant exercise intensity, different values of *λ*.

To illustrate the effects of different cardiovascular condition on heart rate kinetics, Figs 15 and 16 present the on- and off-transient heart rate curves as simulated for subject 1 and another hypothetical subject of the same *HR*_*min*_ and *HR*_*max*_ but different cardiovascular condition level *λ* = 0.55. The exercise intensity was assumed to be constant and equal to *v* = 14.5 *Km*/*hr* (moderate for subject 1, heavy for the hypothetical subject) and the initial heart rate was assumed equal for both subjects *HR*(0) = 70 *beats/min*.

In the graphs presented in Figs 15 and 16 it is worth observing:

- the difference in slope, such that the better the cardiovascular condition the fastest the heart rate kinetics (as is supported by the literature in the area of exercise physiology)
- the significant slow component effect in the on-transient heart rate kinetics of the hypothetical subject

## Conclusions

A powerful improved model has been derived as a result of modifying the dynamical systems model of [6, 21]. The new model is able not only to provide important information regarding an individual’s cardiovascular condition but to also simulate and predict heart rate kinetics for any given exercise intensities.

It is worth emphasizing the impressive accuracy of the model’s prediction regarding resting heart rate values. This way no previous measurements of the *HR*_*min*_ are necessary in order for the model to be fit, as is the case with the existing models. The term *HR*_*min*_ can be substituted by equations (6) or (7) in all relations of the model where it appears. This gives a big advantage to the model, as the experimental estimation of *HR*_*min*_ is in general not an easy task, requiring experience, effort and concentration.

## Supporting Information

S1 Dataset HR data. First column: time (in seconds), second column: heart rate (in beats/min). Exercise A: constant velocity 13.4 *Km*/*hr*; Exercise B: recovery after exercise A; Exercise C: constant velocity 14.4 *Km*/*hr*; Exercise D: recovery after exercise C; Exercise E: constant velocity 15.7 *Km*/*hr*; Exercise F: recovery after exercise E; Exercise G: constant velocity 17.0 *Km*/*hr*; Exercise H: recovery after exercise G.
(RAR)
